# Antiepileptic Drug Discovery and Development: What Have We Learned and Where Are We Going?

**DOI:** 10.3390/ph3092884

**Published:** 2010-09-01

**Authors:** Aaron C. Gerlach, Jeffrey L. Krajewski

**Affiliations:** Icagen, Inc., 4222 Emperor Blvd, Durham, NC 27703, USA; E-Mail: jkrajewski@icagen.com (J.L.K.)

**Keywords:** epilepsy, antiepileptic, AED, seizure, anticonvulsant

## Abstract

Current marketed antiepileptic drugs (AEDs) consist of a variety of structural classes with different mechanisms of action. These agents typically have non-overlapping efficacy and side-effect profiles presenting multiple treatment options for the patient population. However, approximately 30% of seizure sufferers fail to respond to current therapies often because poorly tolerated side-effects limit adequate dosing. The scope of this review is to summarize selected advances in 2^nd^ and 3^rd^ generation AEDs as well as compounds in development with novel mechanisms of action.

## 1. Introduction

Epilepsy is currently defined as the occurrence of at least one seizure with an enduring alteration in the brain structure or function that increases the likelihood of future seizures [1]. Seizures are broadly classified as either generalized or partial depending on whether they involve widespread bilateral cortical regions at the outset or originate from a discrete focal area. This designation is based on both outward symptoms and EEG patterns [2]. Worldwide close to 50 million people are afflicted with epilepsy and even in mild cases the disease can have a severe impact on quality of life [3]. Although methods such as surgery, vagal nerve stimulation and dietary changes have been employed to treat epilepsy, antiepileptic drugs (AEDs) remain the most widely utilized treatment strategy.

Currently over twenty marketed AEDs can be classified as first line therapeutics. No one treatment strategy dominates in terms of efficacy and none are without side effects. In selecting an AED treatment option, doctors and patients consider a myriad of factors including efficacy and side effect profile as well as cost and dosing convenience. A typical therapeutic strategy is to optimize the use of a single AED, given that ~60% of patients have become seizure free using this approach [4]. As a second line approach, concurrent treatment with more than one AED is employed. Unfortunately, only 5% of patients who fail to respond adequately to monotherapy experience long term freedom from seizures using polytherapy [5,6]. The remaining patients are treatment-resistant in that seizures are not adequately controlled. 

The major goal of AED therapy is to allow patients to maintain a normal lifestyle by complete control of seizures with minimal side effects. Ideally this would be obtained using a convenient dosing regimen with minimal limitations on concurrent medications. There is no doubt that tremendous progress has been made toward this goal since the serendipitous discovery in 1912 of phenobarbital, the first widely used anticonvulsant. The subsequent surge in AEDs such as benzodiazepines, valproic acid and phenytoin was a direct consequence of the development of animal seizure models amenable to compound screening. However many of the AEDs discovered in this manner are associated with dose-limiting side effects, adverse reactions and the risk of toxicity through drug-drug interactions. The realization that these early compounds could be further optimized for tolerability and/or pharmacokinetic properties has sparked rational drug design efforts for development of subsequent 2^nd^ and 3^rd^ generation AEDs. 

The scientific understanding of seizure pathogenesis and propagation is far from complete and the mechanism of action of most available AEDs is either unknown or involves multiple interactions. Despite these knowledge gaps, it is generally recognized that AEDs either modulate voltage-gated ion channels, facilitate inhibitory neurotransmission, attenuate excitatory neurotransmission and/or modulate synaptic release [7]. This knowledge, coupled with genetic associations with epilepsy, has facilitated a more recent target-based approach to novel AEDs. The scope of this review is to use selected examples to describe rational design approaches utilized by the pharmaceutical industry to develop new and improved AEDs. The review does not cover every AED in development, nor compounds that have been discovered solely by screening in newer *in vivo* models. Examples of rationally designed 2^nd^ generation and target-based compounds with unique efficacy profiles, improved therapeutic indices, lower risks for toxicity or drug-drug interactions are presented. Current trends and challenges in developing new AEDs are also discussed. 

## 2. Improvements in Currently Marketed AEDs

The majority of second generation compounds are made in an effort to enhance the clinical efficacy of an original AED. This can be accomplished by improving upon a pharmacophore common to many clinically effective AEDs, enhancing the pharmacokinetic parameters of the active compound through improvement of absorption or by reducing metabolism. This section describes the development of three structural classes of AEDs using these methods: retigabine, valproic acid analogs and carbamazepine analogs. 

### 2.1. 1^st^ Generation: Flupirtin; 2^nd^ Generation: Retigabine

**Figure 1:**
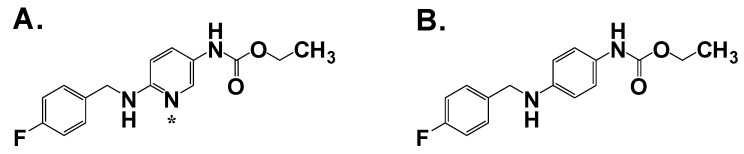
**A**. Flupirtine; **B**. Retigabine. Asterisk denotes basic nitrogen.

Flupirtine (Figure 1A) was first marketed as an analgesic in Europe in 1981. In an effort to better understand its clinical potential, flupirtine was submitted to the Antiepileptic Drug Development program at the NIH where it demonstrated broad efficacy in several antiepileptic models, as well as in a small efficacy study in humans [8]. Through the use of molecular modeling of flupirtine and close analogs, an anticonvulsant pharmacophore was identified. This pharmacophore had significant overlap to several unrelated anticonvulsants already on the market such as diazepam, phenacemide and phenobarbital [9]. Using this knowledge, and the fact that flupirtine was known to be well tolerated in humans, an effort was made to develop an analog with better anticonvulsant activity. Out of this process, a despyridyl analog of flupirtine was made, called D-21329 (retigabine, Figure 1B). The removal of the basic pyridyl nitrogen generated a compound with improved anticonvulsant properties, but decreased its analgesic effects [10]. In light of this improved pre-clinical profile, retigabine was developed clinically as an anticonvulsant. 

Retigabine was active in a battery of anticonvulsant animal models, with lower ED_50_ values in PTZ (pentylenetetrazol), picrotoxin and MES (maximal electroshock) than most standard anticonvulsants, but not active against bicuculline- and strychnine-induced seizures [10]. Of the standard anticonvulsants tested, only valproate had a similar profile to retigabine. Retigabine was also anticonvulsant in the DBA/2 mouse, a model for audiogenic susceptible seizures, with an ED_50_ of4.6 mg/kg p.o. and effectively prevented seizures in models, such as the lamotrigine-resistant kindled rat and the 32 and 44 mA 6 Hz mouse, in which many conventional anticonvulsants are inactive [11]. Most importantly, retigabine did not show signs of tolerance with up to 20 days of treatment in the MES model, a significant problem with many anticonvulsants prescribed today [10]. To calculate the protective index (PI), and thus define whether the apparent anticonvulsant activity was due to motor impairment, the dose of retigabine required to increase the threshold above control 50% (TID_50_) was determined for both MES (anticonvulsant activity) and rotorod (motor impairment). Retigabine had a reported PI of 12.8 in mice and 13.8 in rats [10], which compared favorably to phenytoin, clonazapam, valproate and diazepam, while being lower than the PI for carbamazepine and phenobarbital.

Retigabine was evaluated as an adjuvant therapy in a Phase II trial in 396 patients suffering from partial-onset seizures who were inadequately controlled by 1–2 AEDs. Retigabine significantly reduced partial seizure frequency *vs.* placebo in a dose-related manner in all treatment arms [12]. Retigabine was also evaluated in two Phase III trials where the criteria for patient enrollment, 538 in total, were ≥4 seizures/month while taking 1–3 AEDs. These studies confirmed the dose-dependent efficacy for retigabine was between 600–900 mg/day [13]. Adverse events reported for retigabine in all studies were consistent with most AEDs: dizziness, somnolence and fatigue. These adverse events were dose-related, with 14% and 26% of the enrolled patients withdrawing from the study at the 600 mg/day and 900 mg/day respectively [13]. Given that patient tolerance of the therapy was contingent on titration/dose management, a follow-on to retigabine may focus on a slow-release formulation to minimize these issues. 

### 2.2. 1^st^ Generation: Valproic acid (VPA); 2^nd^ Generation: valnoctamide (VCD), propylisopropylacetamide (PID), 2,2,3,3-tetramethylcyclopropylcarboxcylic acid (TMCA), N-methyl-2,2,3,3-tetramethylcyclo-propanecarboxamide (MTMCD), 2,2,3,3 tetramethylcyclopropanecarbonylurea (TMCU), valrocemide

VPA was serendipitously discovered in 1963 in the PTZ seizure model as a vehicle used in the study of putative antiseizure compounds [14]. Since that discovery, VPA has become one of the most frequently prescribed AEDs worldwide, and has additionally demonstrated therapeutic benefit in other CNS conditions such as bipolar disorder [15] and migraine [16]. The precise molecular target of VPA is unknown, however VPA is active against voltage-dependent Na^+^ and Ca^2+^ channels, multiple enzymes involved in GABA metabolism, and down-regulates phospholipase A2 and consequent arachidonic acid production [17]. VPA has shown efficacy in humans for absence, partial and generalized tonic-clonic seizures. The major limitations of VPA are its potential for teratogenicity, hepatoxicity and drug-drug interactions, all of which narrows its protective index and complicates the dosing regimen. 

**Figure 2:**
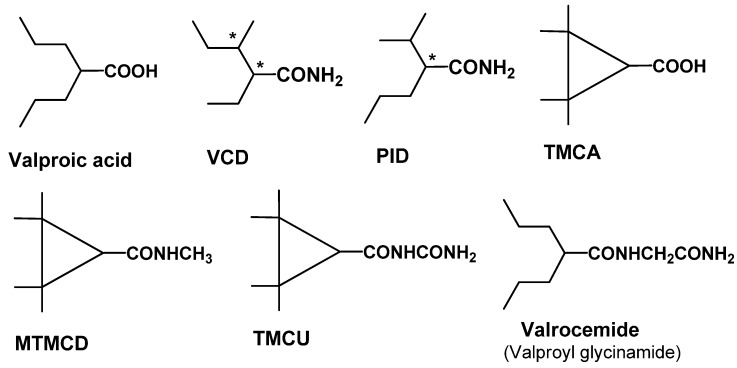
Structure of valproic acid and key 2^nd^ generation analogs. Chiral centers are denoted with an asterisk.

Recent advances in follow-on compounds have been made by identifying and modifying structural elements that correlate with adverse effects *in vivo* [18]. The structures of these key derivatives are shown in Figure 2. VCD and PID represent two such 2^nd^ generation compounds that present a lower risk for teratogenicity compared to VPA. In both compounds, the carboxylic acid has been changed to an amide or acetamide group and the nature of the carbon branching modified. An additional advantage of VCD and PID, unlike various other VPA analogs, is that they do not require metabolic conversion to the acid form for efficacy. Both compounds have a broad range of anticonvulsant activity including activity in hippocampal kindled rats and in the 6 Hz psychomotor seizure model in mice [19,20]. These encouraging results suggest that these amides could find utility in patients with treatment-resistant epilepsy. 

**Figure 3:**
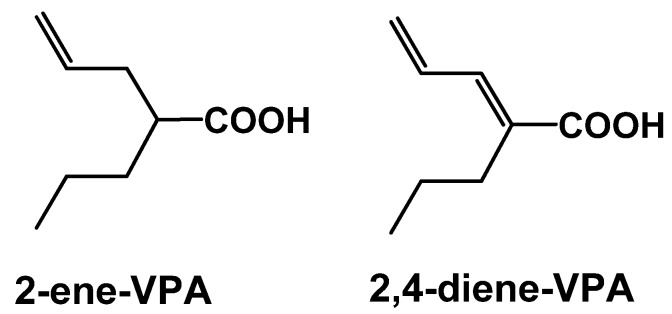
Structures of known hepatotoxic valproic acid metabolites: 2-ene-VPA and 2,4-diene-VPA.

Structural features of VPA that give rise to an increased risk of hepatotoxicity have been identified as well. VPA metabolites with a terminal double bond, such as 4-ene-VPA or 2,4-diene VPA, have been linked to hepatotoxic activity and are shown in Figure 3. The discovery of tetramethyl-cyclopropyl (TMC)-analogs provide promise in addressing this issue. TMCA is a cyclopropyl analog of VPA with two quaternary carbon atoms in the β-position of the carboxylic group. As a result, TMCA cannot be biotransformed into a hepatotoxic metabolite with a terminal double bond. Unfortunately, TMCA is a rather weak anticonvulsant in animal models. However, its amide derivatives, MTMCD and TMCU are efficacious, non-hepatotoxic and non-teratogenic resulting in a 10-fold improvement in protective index relative to VPA. These compounds show broad range anticonvulsant activity in animal models and are in the preclinical phase of discovery [21].

The most advanced VPA 2^nd^ generation compound is valrocemide (valproyl glycinamide), in which a major inhibitory neurotransmitter has been linked with the goal of improving efficacy. Previous attempts had been made with GABA, taurine and glycine conjugates. However, due to poor CNS penetration these compounds were inactive as anticonvulsants [22]. To increase brain exposure taurinamide and glycinamide VPA conjugates were synthesized that could then be biotransformed to either the valproyl taurine or valproyl glycine metabolite Both compounds were active in preclinical rodent seizure models with valrocemide having the most favorable overall profile [23]. In prelimary open-label adjunctive therapy studies in patients with epilepsy, valrocemide was well tolerated at maintenance dosages up to 2,000 mg/twice daily [24]. As most recently reported at the Ninth Eilat Conference, valrocemide is currently in Phase IIa clinical development [13]. 

### 2.3. 1^st^ Generation: Carbamazepine; 2^nd^ Generation: Oxcarbazepine; 3^rd^ Generation: Eslicarbazepine acetate (ESL)

Carbamazepine (CBZ) was first approved in 1968 for use in partial-onset and generalized tonic-clonic seizures. The efficacy of CBZ is attributed to inhibition of the inactivated state of voltage-dependent Na^+^ channels, resulting in a preferential inhibition of high frequency neuronal firing. CBZ continues to be used as a first line AED despite its major limitations of generation of an active metabolite, carbamazepine-10,11-epoxide, and auto-induction of CYP enzymes leading to the potential for drug-drug interactions. The 2^nd^ and 3^rd^ generation approaches to CBZ have been targeted to resolve these issues, while retaining the efficacy profile for generalized and partial seizures. 

**Figure 4:**
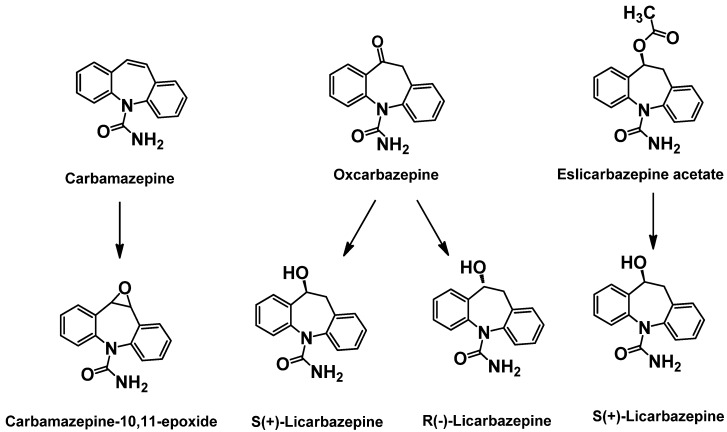
Structures of carbamazepine, new generation analogs and key metabolites.

Unlike CBZ, the follow on compound oxcarbazepine (OXC) does not undergo inducible cytochrome CYP3A4-mediated oxidative metabolism. Instead OXC is biotransformed by 10-keto reduction to the active monohydroxy derivative, 10-hydroxycarbazepine, also known as licarbazepine. The conversion of OXC to licarbazepine is stereoselective with the *S* enantiomer having an AUC exposure five-fold that of the *R* enantiomer [25]. Therefore, *S*-licarpazepine is considered as the major active species following OXC treatment and has led to 3^rd^ generation approaches to maximize the exposure of *S*-licarpazepine. Structures of these parent compounds and key metabolites are shown in Figure 4. 

Eslicarbazepine acetate (ESL) is a pro-drug that is >95% converted to *S*-licarbazepine. The bioavailability is 16% greater compared to an equivalent dose of OXC [26]. In a recent double-blind placebo controlled trial, ESL showed improvements over both CBZ and OXC in the potential for once daily dosing. In this trial, subjects receiving either once or twice daily dosing of ESL (800 mg or 1200 mg) showed a statistically significant reduction in number of seizures [27]. Furthermore, the once daily group had similar responses to those treated twice daily with ESL. Interestingly, 60% of the patients enrolled in the Phase III trials were not fully treated with CBZ yet still benefited from ESL. These results suggest that modest increase in plasma exposure of *S*-licarbazepine compared to that from OXC may have clinical relevance. ESL is currently approved in Europe (Zebinix) as an adjunctive therapy in adults with partial-onset seizures. An NDA for the same indication was submitted for FDA approval in 2009 and is currently under review. 

## 3. Mechanism Based Drug Discovery

The typical path of first generation AED drug discovery was to identify active compounds using rodent seizure models (i.e. PTZ, MES, kindling, *etc*). While this strategy has been successful for identifying several novel classes of AEDs, the exact mechanism of drug action was unclear because the molecular target was unknown. Identifying the molecular target for AED activity typically allows chemists to more efficiently optimize multiple properties based on target potency and selectivity prior to advancing compounds into animal studies. The following section describes the identification and/or development of several AEDs through a mechanism-based approach. For example, brivaracetam, a structurally related second generation AED to levetiracetam, was discovered using a high-throughput screen on the novel levetiracetam-binding site (LBS) present on SV2A. Moreover, pregabalin, a structurally similar second generation AED to gabapentin, was identified as a potent and selective ligand to the α2δ-subunit of voltage-gated calcium channels. A third novel AED in clinical development, ICA-105665, was discovered by screening novel compounds on KCNQ2/3 potassium channels, the molecular target of retigabine (see Part 1). 

### 3.1. Molecular Target: SV2A—1^st^ Generation: Levetiracetam; 2^nd^ Generation: Brivaracetam

Levetiracetam was identified using audiogenic seizure-susceptible mice [28] and has proven to be quite effective in treating partial-onset seizures, although it is also used as an adjuvant with other AEDs. The success of levetiracetam as a novel AED prompted researchers to better characterize it in pre-clinical animal models of epilepsy. Although levetiracetam is active in the kindling models of epilepsy [29] as well as in the 6-Hz and absence epilepsy models [30], it is not active in the traditional PTZ and MES seizure models [31]. In order to identify new and improved compounds with potentially greater therapeutic potential, an effort was undertaken to identify the molecular target for levetiracetam.

**Figure 5:**
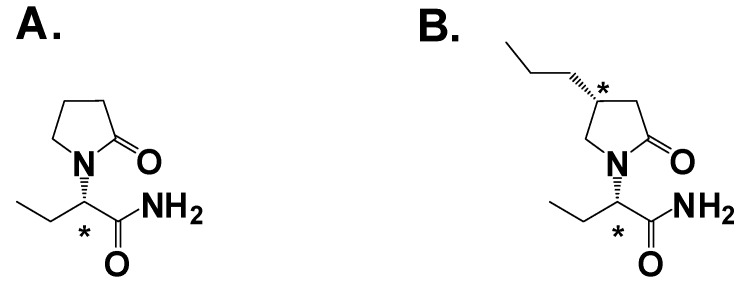
**A.** Levetiracetam; **B.** Brivaracetam. Asterisk denotes a chiral center.

The structure of levetiracetam contains a chiral center (Figure 4A). Experiments using both enantiomers in audiogenic seizure-susceptible mice showed the (*S*)-isomer was 150-fold more potent than the (*R*)-isomer, strong evidence that the molecular target was a protein [28]. Using a radiolabled analog of levetiracetam scientists observed specific binding to a region in the brain [32]. Optimization of the binding assay led to the identification of the binding target as a ubiquitous synaptic vesicle membrane protein, hypothesized to be SV2A [33]. The role of SV2A in neurotransmission is believed to involve priming of the vesicles in quiescent neurons to facilitate low-frequency neurotransmission [34]. The SV2A hypothesis was confirmed by two studies: First, SV2A knockout mice lacked specific [H^3^]-levetiracetam binding [35]. Second, there is a high correlation with *in vivo* efficacy and binding affinity to SV2A in audiogenic induced seizures and corneal kindling, as well as absence seizures for levetiracetam and analogs [36]. Thus identification of the SV2A molecular target and the development of a high-throughput binding assay allowed the screening of ~13,000 compounds and the discovery of brivaracetam (Figure 4B), which has a binding affinity 13-fold greater than levetiracetam [37]. Brivaracetam demonstrated broader activity in animal models [38] but a lower therapeutic index, or TI (the ratio of the ED_50_ for seizure protection in audiogenic seizure-susceptible mice to the TD_50_ of motor impairment on rotorod) [29,38]. 

In a Phase II trial with 208 patients suffering from refractory partial seizures, brivaracetam dose-dependently reduced the frequency of seizures with an adverse event profile indistinguishable from that of placebo [39]. Currently, brivaracetam is in three Phase III randomized, double-blind, muticenter and multinational studies to evaluate its safety and efficacy. Two of these studies will cover 12 weeks of treatment for adults with partial-onset seizures that are inadequately controlled by up to two AEDs. The third study will be a 16 week study in patients with partial-onset or primary generalized seizures inadequately controlled with up to three AEDs. Early results indicate brivaracetam was well tolerated in all studies, and reached statistical significance in one study in reducing seizure frequency as an adjuvant therapy [13].

### 3.2. Molecular Target: α2δ voltage-gated calcium channel subunit—1^st^ Generation: Gabapentin; 2^nd^ Generation: Pregabalin

Gabapentin (Figure 5A) was first approved by the FDA in 1994 as an adjuvant therapy for partial onset seizures. Although the majority of its sales are for neuropathic pain indications, it has enjoyed tremendous success as a widely prescribed AED. Gabapentin has efficacy in a broad array of preclinical models for epilepsy and neuropathic pain. Originally designed to be a GABA-mimetic, surprisingly gabapentin has not demonstrated any activity on GABA_A_, GABA_B_ or GAT-1 transporters. However, gabapentin does have an unusually high affinity for the system L amino acid transporter, which allows endogenous amino acids to cross the blood-brain barrier [40]. Studies using [^3^H]-gabapentin demonstrated high-affinity binding to a novel protein in porcine brain membrane homogenate [41] later identified as the α2δ-subunit of the voltage-gated calcium channel [42]. The anticonvulsant efficacy of gabapentin and analogs are highly correlated with α2δ binding affinity [43]. For example, (*S*)-3-aminomethyl-5-methylhexanoic acid (Figure 5B, pregabalin) was identified in a binding assay as a potent α2δ ligand, which conferred 100% protection in mice from audiogenic-induced seizures at 30 mg/kg p.o. [44]**.** Pregabalin was approved for use as an adjuvant for partial onset seizures in 2004.

**Figure 6:**
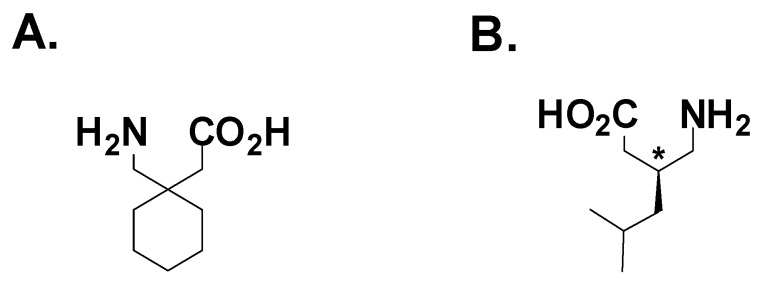
**A.** Gabapentin; **B.** Pregabalin.

The exact mechanism by which modulation of α2δ can confer anticonvulsant efficacy has not been fully defined. Studies with gabapentin have demonstrated a decrease in presynaptic calcium entry leading to a reduction in neurotransmitter release [45,46]. Although direct inhibition of voltage-gated calcium channels with gabapentin have yielded equivocal results [47,48,49]. Chronic gabapentin exposure (>40 hours.) of cells co-expressing α2δ with Cav2.1 or Cav2.2 decreased calcium current density due to a significant reduction of plasma membrane expression of the α2δ protein and its associated voltage-gated calcium channel [48]. This gabapentin-mediated decrease in calcium channel surface expression was not observed in cells expressing a mutated form of α2δ that removed the gabapentin binding site. An SAR study of gabapentin and close analogs demonstrated the requirement of high affinity binding to α2δ to enable protection of DBA/2 mice from audiogenic-induced seizures [44]. Based on the relationship of α2δ affinity and antiepileptic efficacy, identifying the function of the α2δ-subunit may allow the development of more novel AEDs in the future 

### 3.3. Molecular Target: M-current (KCNQ2/Q3, Kv7.2/Kv7.3)—ICA-105665 (Icagen)

The success of retigabine as a novel anticonvulsant led to various groups determining its mechanism of action (see Part 1). In a hippocampal slice preparation, retigabine suppressed epileptiform activity induced by the application of 4-aminopyridine, an inhibitor of voltage-gated potassium channels [50]. This led Rundfeldt to investigate if retigabine exerted its anticonvulsant effects through a direct effect on voltage-gated potassium channels. Retigabine was able to induce a rapid non-desensitizing outward current, determined to be a potassium conductance, in the neuronal cell line NG-108-15 [51]. The retigabine-induced current was not blocked by extracellular application of 4-aminopyridine or glibenclamide (K_ATP_ blocker), but was sensitive to 10 mM Ba^2+^. None of the standard anticonvulsants (carbamazepine, valproate, phenytoin) induced an outward potassium current in NG-08-15 cells similar to that which was seen with retigabine [51]. These data suggest that a retigabine-induced increase in potassium conductance may be a novel anticonvulsant mechanism of action. 

The molecular target for retigabine was identified by comparing the properties of the current induced by retigabine (non-desensitizing, linear current-voltage relationship over a wide range) and its pharmacology (weakly tetraethylammonium (TEA) sensitive, blocked by Ba^2+^, insensitive to 4-aminopyridine) to the properties and pharmacology of other known potassium channels. Only the M-current potassium channel current matched all of these observations. The molecular correlate of the M-current has been identified as the KCNQ2 (Kv7.2) and KCNQ3 (Kv7.3) subunits, which form a heteromultermeric channel [52]. Using heterologous expression of KCNQ2/Q3 in Xenopus oocytes [53] and mammalian cells [54], retigabine induced outward currents with similar properties to those seen in NG-108-15 cells and this induced current was sensitive to known M-current modulators. 

Prior to the identification of KCNQ2/Q3 as the molecular target for retigabine, there have been numerous publications that lead to the conclusion that the M-current plays a significant role in neuronal excitability, and thus may be an attractive target for a novel AED agent. First, mutations in KCNQ2/Q3 channels are thought to underlie a rare form of neonatal epilepsy [55,56]. Second, KCNQ2/Q3 are located both pre- and postsynaptically in brain regions that are important for controlling neuronal excitability [57]. Finally, inhibition of the M-current with muscarinic agonists or linopirdine causes neuronal hyperexcitability *in vitro* and is proconvulsant *in vivo* [58,59]. In total, these findings provide scientists strong biological rationale to pursue KCNQ2/Q3 as an antiepileptic target and retigabine as a valuable tool.

Confirmation that KCNQ2/Q3 is the molecular target for retigabine enabled researchers to develop a high throughput assay to screen a large compound library to identify novel KCNQ2/Q3 activators. Using a neuroblastoma cell line known to express KCNQ2/Q3 channels, agonists or activators were identified when a hyperpolarization of the membrane was detected with a voltage-sensitive dye [60]. One compound identified using this method was ICA-27243. Electrophysiological characterization on KCNQ subtypes demonstrated that ICA-27243 was selective for the KCNQ2/Q3 channel over other KCNQ subtypes [60]. In the same assays, retigabine exhibited no selectivity among KCNQ subunits expressed in neurons (e.g., KCNQ3/Q5), making ICA-27243 the first identified KCNQ2/Q3 selective activator. ICA-27243 was also selective over other ion channels that are known AED targets, such as voltage-gated sodium and calcium channels, as well as GABA-activated chloride channels. In the MES mouse epilepsy model, ICA-27243 had an ED_50_ of 8.4 mg/kg when dosed orally. Given the selectivity of ICA-27243, this finding demonstrated that opening KCNQ2/Q3 channels alone is sufficient for efficacy in a preclinical model of epilepsy [60]. 

Using a combination of screening, animal testing and medicinal chemistry, other KCNQ channel selective chemotypes were identified. One such compound, ICA-105665, has broad efficacy in preclinical models of epilepsy and both inflammatory and neuropathic pain. In animal studies it has a PI greater than >100 for epilepsy (ED_50_ for rat MES is 0.9 mg/kg and TD_50_ for rotorod is >100 mg/kg) and in Phase I clinical trials it has been generally well tolerated at all doses tested with no dose limiting side-effects [61]. Moreover, ICA-105665 reduced the photoparoxysmal electroencephalographic (EEG) response in epilepsy patients with photosensitivity using well-established standardized methods [62,63]. The results of this study were presented at the EILAT X Conference [64]. Further clinical development of ICA-105665 may include the study of higher doses in patients with photosensitive epilepsy, as well as larger clinical trials in patients with treatment-resistant, partial-onset epilepsy. 

## 4. Conclusions and Future Directions

Although dozens of antiepileptic drugs have been marketed for the treatment of epilepsy, there remains a large unmet medical need for the development of better therapies for drug-resistant epilepsy patients. Currently, there is no single drug of choice in treating all types of seizures and no drug that has substantially lowered the percentage of patients who are drug-resistant. The inability of AEDs to adequately treat all patients suffering from epilepsy is probably due to several factors. First, many 1^st^ generation AEDs were originally identified by random screening of compounds in acute animal seizure models. Epilepsy is a complex and heterogeneous disorder in the human population and it is unlikely that acute animal models can broadly predict the therapeutic potential of investigational drugs. Second, many of the 2^nd^ generation AEDs are very close analogs of the original compounds. While perhaps having an improved side-effect and/or pharmacokinetic profile, these analogs likely have similar mechanisms of action, which would be unlikely to broaden their therapeutic potential. Finally, while early AEDs may be potent anticonvulsants, many have dose-limiting toxicity and/or unacceptable side-effects, which prevent achieving adequate brain levels to completely control seizures. Without a thorough understanding of the mechanism of these older drugs, it has been difficult and laborious to eliminate unwanted properties by screening for improved compounds in animal models.

The path to future AED development lies in a better understanding of the pathology of epilepsy, which will lead scientists to identify novel targets for AED development and to develop animal models that better recapitulate the human phenotype. An example of the former is the report that identified a mutation in KCNQ2/Q3 (Kv7.2/Kv7.3) as responsible for the formation of a rare form of neonatal epilepsy [55,56]. KCNQ2/Q3 as a target for AED development was further strengthened when it was reported that retigabine, a broadly active AED in animal models, was a selective opener of KCNQ2/Q3 and may explain its mechanism of action in the clinic [53,54]. Since then other structurally unrelated compounds that are selective openers of KCNQ2/Q3 have been identified and demonstrate activity in animal models [60] and are in clinical development. A better understanding of the pathogenesis of epilepsy will also aid in developing improved animal models that may increase the likelihood of predicting clinical success for a novel investigational drug. There are several human genetic mutations that appear to lead to an epilepsy phenotype, (for review see [65]) which can form the basis for the generation of animal models. An example is the severe myoclonic epilepsy of infancy (SMEI) mouse, which possesses a phenotype very similar to that found in humans suffering from SMEI [66,67]. The genotype for this animal model is a single-mutation in the SCN1A (Nav1.1) sodium channel which leads to a loss of function and thus to decreased sodium current density [68]. This mutation is relevant to AED development because lamotrigine, an AED used to treat generalized and partial seizures, acts as a proconvulsant clinically in infants with SMEI [69]. As we expand our knowledge of the pathogenesis of epilepsy and researchers develop more animal models which recapitulate the human phenotype, we may see an increased number of AEDs targeted for specific seizure disorders. 

Finally, future efforts to discovery novel AEDs are likely to focus on mechanism-driven discovery of novel compounds, followed by informed animal testing in relevant drug-resistant animal models. Several recent successes (pregabalin, brivaracetam) have shown that knowledge of the mechanism of action gives the developer a significant advantage in improving efficacy through increased target potency and selectivity, thereby lowering the potential for dose-related side effects. However, to date even recent advances have not significantly reduced the size of drug-resistant seizure population [5,6]. It is the hope that new generation AEDs with novel mechanisms will increase the likelihood for success in treating a heterogeneous patient population including those patients suffering from drug-resistant forms of epilepsy.
